# Clinicopathologic factors and preoperative ultrasonographic characteristics for predicting central lymph node metastasis in papillary thyroid microcarcinoma: a single center retrospective study

**DOI:** 10.1016/j.bjorl.2020.05.004

**Published:** 2020-06-04

**Authors:** Liang Jiwang, Luo Yahong, Liang Kai, Huang Bo, Zhao Yuejiao, Wang Haotian, Yu Tao

**Affiliations:** a Cancer Hospital of China Medical University, Liaoning Cancer Hospital & Institute, Department of Head and Neck Surgery, Shenyang, China; b Cancer Hospital of China Medical University, Liaoning Cancer Hospital & Institute, Department of Medical Imaging, Shenyang, China; c Cancer Hospital of China Medical University, Liaoning Cancer Hospital & Institute, Department of Pathology, Shenyang, China; d Grade 2016 Clinical Medicine Class 5, The First Clinical College, Dalian Medical University, Shenyang, China

**Keywords:** Papillary thyroid microcarcinoma, Central lymph node metastasis, Predictive factors, Ultrasound, Nomogram

## Abstract

**Introduction:**

The treatment of papillary thyroid microcarcinoma remains controversial. Central lymph node metastasis is common in papillary thyroid microcarcinoma and it is an important consideration in treatment strategy selection.

**Objective:**

The aim of this study was to investigate clinicopathologic risk factors and thyroid nodule sonographic characteristics for central lymph node metastasis in papillary thyroid microcarcinoma.

**Methods:**

We retrospectively reviewed the data of 599 papillary thyroid microcarcinoma patients who underwent surgery from 2005 to 2017 at a single institution. Univariate and multivariate analyses were used to identify the clinicopathologic factors and preoperative sonographic features of central lymph node metastasis. A receiver-operating characteristic, ROC curve analysis, was performed to identify the efficacy of ultrasonographic features in predicting central lymph node metastasis. A nomogram based on the risk factors was established to predict central lymph node metastasis.

**Results:**

The incidence of central lymph node metastasis was 22.4%. The univariate and multivariate analyses suggested that gender, age, multifocality, extrathyroidal invasion, and lateral lymph node metastasis were independent risk factors for central lymph node metastasis. The univariate and multivariate analyses revealed that nodular shape, margin, and calcification were independently associated with central lymph node metastasis. The ROC curve analysis revealed that the combination of shape, margin and calcification had excellent accuracy in predicting central lymph node metastasis. The nomogram was developed based on the identified risk factors for predicting central lymph node metastasis, and the calibration plot analysis indicated the good performance and clinical utility of the nomogram.

**Conclusions:**

Central lymph node metastasis is associated with male gender, younger age (<45 years), extrathyroidal invasion, multifocality and lateral lymph node metastasis in papillary thyroid microcarcinoma patients. The ultrasongraphic features, such as irregular shape, ill-defined margin and calcification, may improve the efficacy of predicting central lymph node metastasis. Surgeons and radiologists should pay close attention to the patients who have these risk factors. The nomogram may help guide surgical decision making in papillary thyroid microcarcinoma.

## Introduction

Thyroid carcinoma is the most common form of neoplasia of the endocrine system, and its incidence rate is increasing by an average of 4.5% per year from 2007 to 2011. Papillary thyroid carcinoma (PTC) is the most common histological subtype of thyroid cancer, accounting for 80% of all thyroid malignancies.^1^ According to the World Health Organization classification system, papillary thyroid microcarcinoma (PTMC) is defined as a PTC measuring ≤1.0 cm in its greatest dimension.^2^ In recent years, the widespread use and the technical improvement of ultrasound (US) and US-guided fine-needle aspiration biopsy (FNAB) contributed to an increase in the rate of preoperative diagnosis of PTMC.^3^, ^4^ Some studies showed that the percentage of PTMC among all thyroid cancer in the last decade reached 39.5% even 42.0%.^5^, ^6^

Although PTMC usually follows an indolent course and has an excellent prognosis, lymph node metastasis (LNM) is present in 17–64% of PTMC patients and 80% of recurrent PTMC patients.^7^, ^8^ Many authors consider that LNM was an important risk factor. A large population-based study suggested that differentiated thyroid cancer with LNM resulted in a 3 fold higher disease-related mortality rate.^9^ Central lymph node metastasis (CLNM) in PTC cancer is common. Early detection of CLNM plays an important role in deciding the therapeutic choice. For the management of central lymph node (CLN), the American Thyroid Association guidelines recommend prophylactic central lymph node dissection (pCLND) may be performed in clinically node-negative PTC patients, particularly those with extrathyroidal extension or with tumor size >4 cm.^10^ However, because the recommendation using only two pathologic features might be insufficient to predict subclinical CLNM, pCLND has been performed routinely or according to the clinician's personal preference. Moreover, pCLND may increase morbidities such as recurrent laryngeal nerve injury and hypoparathyroidism. Thus, surgeons need to better understand the predictive risk factors of CLNM when deciding whether to perform CLND.

Currently, preoperative US are the first-line method of examining LNM in PTC patients. Metastatic lymph nodes often appear with calcification, cystic necrosis, hyper-echogenicity, a round shape, peripheral or mixed vascularity and the absence of an echogenic hilum.^11^, ^12^, ^13^ However, the ability of preoperative US to identify CLNM is limited due to the overlying thyroid gland, and these lymph nodes do not often appear abnormal on preoperative imaging or by inspection at the time of surgery. Thus, US may have low predictive accuracy for CLNM before surgery. In addition, the usefulness of US when determining CLNM in PTMC is still controversial.^14^, ^15^, ^16^ Considering that prediction of CLNM is important for potentially altering the clinical decision-making, many authors have studied the preoperative ultrasonographic features of CLNM. Currently, improving the accuracy of diagnosis of CLNM before surgery remains a challenge.

Although there have been numerous investigations identifying the predictive clinicopathologic factors for CLNM, the results are inconsistent. Whether US are a good choice for CLNM evaluation in PTMC remains unclear. Thus, identification of clinicopathologic factors and thyroid nodule sonographic features for predicting CLNM is likely to facilitate optimal therapeutic decision in PTMC. In this study, we retrospectively examined the incidence and related clinicopathologic features of CLNM in PTMC patients, attempting to reveal the independent predictors. We also evaluated thyroid nodule ultrasonic features associated with CLNM in these patients, which might help to more accurately determine the preoperative status of CLNM. Additionally, we established a nomogram to predict the risk of CLNM preoperative, and these outcomes could assist greatly in decision-making regarding further treatment.

## Methods

Between January 2005 and June 2017, there were 2046 patients who underwent thyroid surgical treatment in a single institution. The present study was accordance with the Declaration of Helsinki and was approved by the Institutional Ethical Committee (approval no. 20170301, 20181207). Patients consent was achieved by informed consent forms.

The inclusion criteria was as follows: (1) patients without previous history of radiation and other head and neck malignant tumors; (2) patients who underwent thyroidectomy and CLND; (3) PTMC had been confirmed by histopathological examination of the surgical specimen; (4) the medical records of patients were complete. The patients who had non-PTC (follicular, medullary and anaplastic), and metastatic carcinoma from other organs were excluded from this study. Patients with previous thyroid or parathyroid surgery were excluded. The patients who did not have a neck US were also excluded. PTMC diagnosis was defined as PTC whose maximum size is ≤1.0 cm according to World Health Organization Standards. After the inclusion and exclusion criteria, 599 PTMC patients were included. The following variables were considered: age at diagnosis, gender, Hashimoto thyroiditis (HT), multifocality, extrathyroidal invasion, bilaterality, type of surgery, CLNM, and lateral lymph node metastasis (LLNM).

In our study, all patients underwent careful history and thorough physical examination. All patients underwent a preoperative examination with a high-resolution US equipped with a 7–12 MHz linear probe. All neck US examinations were performed by radiologists with more than 5 years of experience in thyroid imaging. The patients were placed in a supine position, and US features included number of nodules, nodule location, unilateral or bilateral gland involvement, shape, margin, echogenicity, internal content, calcification, anteroposterior to transverse ratio (A/T), and blood flow. Sensitivity, specificity, positive predictive value (PPV), negative predictive value (NPV), and accuracy for the US features suspicious for CLNM were calculated. The diagnostic accuracy of prediction of CLNM was calculated with receiver operating characteristic (ROC) analysis. The area under the curve (AUC) was estimated from the ROC curve.

Surgical management has been performed according to the American Thyroid Association guidelines 2009 in our institution.^17^ Our study included patients with both therapeutic CLND and pCLND. In the cases herein, pCLND was performed in clinically node-negative patients with extrathyroidal invasion or tumor size larger than 4 cm or according to the surgeon's personal preference. Therapeutic CLND was performed when CLNM was detected during preoperative or intraoperative examination. CLND was performed cranially to the superior artery and pyramidal lobe, caudally to the innominate vein, laterally to the carotid sheaths, and dorsally to the prevertebral fascia. Particular attention was paid to preserving the parathyroid glands and the recurrent laryngeal nerve. Lateral lymph node dissection including levels II–V was performed only in cases with clinically evident LLNM. Surgical treatment consisted of total or near-total thyroidectomy in 213 patients, lobectomy in 43 patients, and lobectomy with isthmusectomy in 343 patients. Comprehensive neck dissections such as radical neck dissection and modified neck dissection were performed in 4 patients, and 3 of these underwent bilateral neck dissection.

Median follow-up period was 32.4 months (range: 6.55–155.23 months). All patients with PTMC were examined at 3, 6 and 12 months after the initial treatment and yearly thereafter, or more frequently according to the clinical course. Patient progress was followed by physical examination, US and CT to identify local thyroid remnant, lymph node and distant metastasis.

Univariate analysis was performed using Pearson's chi-square test and Fisher's exact test as appropriate. Statistically significant results obtained from univariate analysis were submitted to multivariate logistic regression. A nomogram was built using the variables in the final multivariate logistic regression model to predict CLNM. The discriminative ability of the predictive nomogram was assessed by Harrell's Concordance Index (C-index).^18^ C-index has a similar meaning to the AUC. The highest value of C-index is 1, which means perfect discrimination; the lowest value is 0.5, which stands for random discrimination. Calibration was used to compare how well the predicted probabilities from our nomogram matched the actual probabilities of CLNM. For all analyses, only *p*-values <0.05 were considered significant. All statistical analyses were performed using the SPSS 16.0 statistical package (SPSS, Inc., Chicago, IL, USA), and R language software and the RMS package.

## Results

There were 105 males (17.5%) and 494 females (82.5%; ratio 1:4.7). There were 284 patients (47.4%) aged <45 years and 315 (52.6%) aged ≥45 years. The mean maximum tumor size was 0.78 ± 0.20 cm. We found CLNs were confirmed as positive with US before surgery in 105 patients, but postoperative pathology results revealed that 27 patients were positive. US visualized 494 negative nodes before surgery, whereas post-surgical pathology results indicated 107 lymph nodes were positive (Table 1). The sensitivity and specificity of US in evaluating CLNM were 20.1% and 83.2%, respectively.

**Table 1:** US and pathologic results for CLNM.

US	Pathologic diagnosis		
	Positive nodes	Negative nodes	Total
Positive nodes	27	78	105
Negative nodes	107	387	494

Total	134	465	599

The clinicopathological characteristics of the 599 PTMC patients are shown in Table 2. Among all patients, multifocality was found in 140 patients (23.4%), bilaterality in 101 patients (16.9%), extrathyroidal invasion in 99 patients (16.5%). HT coexistent with PTMC was observed in 119 patients (19.9%). CLNM was identified in 134 patients (22.4%), LLNM in 60 patients (10%), and both CLNM and LLNM in 37 patients (6.2%). During lymphadenectomy, one to 28 lymph nodes were removed. The number of LNM varied between 0 and 18.

**Table 2:** Clinicopathologic characteristics of 599 PTMC patients.

Variables	Total
*Gender*	
Male	105 (17.5%)
Female	494 (82.5%)
*Age (years)*	
<45	284 (47.4%)
≥45	315 (52.6%)
*Tumor size (mm)*	
≤5	99 (16.5%)
>5	500 (83.5%)
*Multifocality*	140 (23.4%)
*Bilaterality*	101 (16.9%)
*Extrathyroidal invasion*	99 (16.5%)
*Lymph node metastasis*	
Present	157 (26.2%)
Absent	442 (73.8%)
*Central lymph node metastasis*	
Present	134 (22.4%)
Absent	465 (77.6%)
*Lateral lymph node metastasis*	
Present	60 (10.0%)
Absent	539 (90.0%)
*Hashimoto thyroiditis*	
Present	119 (19.9%)
Absent	480 (80.1%)

The risk factors of PTMC patients with CLNM are shown in Table 3. In PTMC patients, male (*p* = 0.000), age <45 years (*p* = 0.000), multifocality (*p* = 0.025), extrathyroidal invasion (*p* = 0.000), and LLNM (*p* = 0.000) were significantly associated with CLNM. Significant results obtained in univariate analysis were subjected to multivariate logistic regression analysis. Male (OR = 0.355; 95% CI 0.219–0.577; *p* = 0.000), age <45 years (OR = 2.67; 95% CI 1.758–4.055; *p* = 0.000), multifocality (OR = 0.626; 95% CI 0.393–0.997; *p* = 0.049), extrathyroidal invasion (OR = 0.378; 95% CI 0.230–0.621; *p* = 0.000), and LLNM (OR = 0.317; 95% CI 0.129–0.778; *p* = 0.012) remained as independent risk factors of CLNM.

**Table 3:** Univariate and multivariate analysis for CLNM with statistically significant variables.

Variables	Univariate analysis			Multivariate analysis		
	CLNM (+)	CLNM (−)	*p*-Value	OR	95% CI	*p*-Value
*Gender*			0.000	0.355	0.219–0.577	0.000
Male	39 (37.1%)	66 (62.9%)				
Female	95 (19.2%)	399 (80.8%)				
*Age (years)*			0.000	2.670	1.758–4.055	0.000
<45	88 (31.0%)	196 (69.0%)				
≥45	46 (14.6%)	269 (85.4%)				
*Tumor size (mm)*			0.059			
≤5	15 (15.2%)	84 (84.8%)				
>5	119 (23.8%)	381(76.2%)				
*Multifocality*			0.025	0.626	0.393–0.997	0.049
Present	41 (29.3%)	99 (70.7%)				
Absent	93 (20.3%)	366 (79.7%)				
*Bilaterality*			0.093			
Present	29 (28.7%)	72 (71.3%)				
Absent	105 (21.1%)	393 (78.9%)				
*Extrathyroidal invasion*			0.000	0.378	0.230–0.621	0.000
Present	37 (37.4%)	62 (62.6%)				
Absent	97 (19.4%)	403 (80.6%)				
*Lateral lymph node metastasis*			0.000	0.317	0.129–0.778	0.012
Present	37 (61.7%)	23 (38.3%)				
Absent	97 (18.0%)	442 (82.0%)				
*Hashimoto thyroiditis*			0.406			
Present	30 (25.2%)	89 (74.8%)				
Absent	104 (21.7%)	376 (78.3%)				

Preoperative US characteristics were compared in patients with and without CLNM, as presented in Table 4. Univariate analysis revealed that statistical differences in shape (*p* = 0.000), margin (*p* = 0.026), echogenicity (*p* = 0.011), calcification (*p* = 0.000), and A/T (*p* = 0.021) between the two groups. Multivariate analysis showed that shape, margin, and calcification were found to be independent factors indicative of CLNM. The odds ratios of these significant factors were 0.464 (95% CI 0.262–0.823, *p* = 0.009), 1.776 (95% CI 1.063–2.966, *p* = 0.028), 0.130 (95% CI 0.081–0.208, *p* = 0.000), respectively.

**Table 4:** Univariate and multivariate analysis of ultrasonographic characteristics in PTMC patients with CLNM.

Variables	Univariate analysis			Multivariate analysis		
	CLNM (+)	CLNM (−)	*p*-Value	OR	95% CI	*p*-Value
*Number of nodules*			0.218			
<1	88 (24.0%)	278 (76.0%)				
≥2	46 (19.7%)	187 (80.3%)				
*Location*			0.841			
Upper third	25 (22.7%)	85 (77.3%)				
Mid third	41 (19.9%)	165 (80.1%)				
Lower third	60 (24.5%)	185 (75.5%)				
Isthmus	3 (20.0%)	12 (80.0%)				
Multicentric	5 (21.7%)	18 (78.3%)				
*Bilaterality*			0.676			
Present	21 (20.8%)	80 (79.2%)				
Absent	113 (22.7%)	385 (77.3%)				
*Shape*			0.000	0.464	0.262–0.823	0.009
Regular	20 (12.4%)	141 (87.6%)				
Irregular	114 (26.0%)	324(74.0%)				
*Margin*			0.026	1.776	1.063–2.966	0.028
Smooth	33 (16.9%)	162 (83.1%)				
Ill-defined	101 (25.0%)	303 (75.0%)				
*Echogenicity*			0.011	0.621	0.375–1.028	0.064
Markedly hypoechoic	19 (30.2%)	44(69.8%)				
Hypoechoic	108 (23.3%)	356 (76.7%)				
Isoechoic/hyperechoic	7 (9.7%)	65 (90.3%)				
*Internal structure*			0.159			
Solid	124 (22.5%)	428 (77.5%)				
Cystic	1 (100.0%)	0 (0)				
Mixed	9 (19.6%)	37 (80.4%)				
*Calcification*			0.000	0.130	0.081–0.208	0.000
Present	105 (40.2%)	156 (59.8%)				
Absent	29 (8.6%)	309 (91.4%)				
*Aspect ration (A/T)*			0.021	0.802	0.509–1.265	0.343
<1	43 (17.6%)	201(82.4%)				
≥1	91 (25.6%)	264 (74.4%)				
*Blood flow*			0.460			
No	20 (18.0%)	91 (82.0%)				
Little	84 (23.7%)	271 (76.3%)				
Rich	30 (22.6%)	103 (77.4%)				

**Figure 1:**
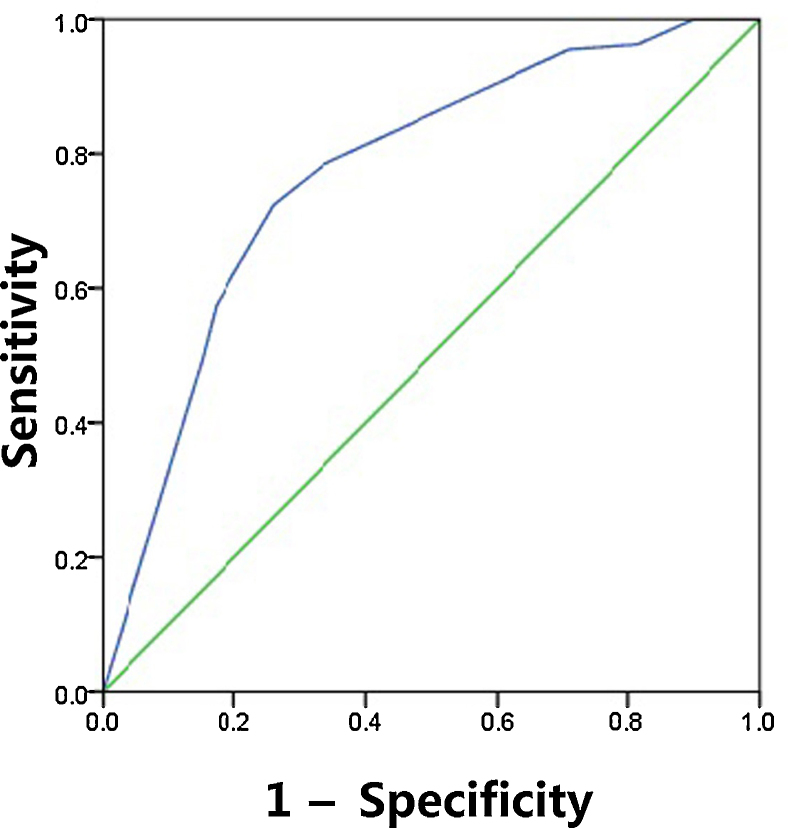
ROC curve for the predictive of preoperative ultrasonographic features. The predictive model of CLNM was accurate and discriminating, with AUC of 0.782. A cut point for prediction of CLNM was defined as a value = 0.468. A predicted probability of 0.468 provided a sensitivity of 72.4% and a specificity of 74.4%.

The sensitivity, specificity, PPV, NPV and diagnostic accuracy of US features are shown in Table 5. The respective sensitivity, specificity, PPV, NPV and accuracy for the prediction of CLNM were as follows: irregular shape 0.303, 0.851, 0.26, 0.876 and 0.426; ill-defined margin 0.652, 0.246, 0.25, 0.831 and 0.439; calcification 0.665, 0.784, 0.402, 0.914 and 0.691. The AUC value of shape and margin was 0.577 and 0.449, respectively, even though they satisfied the *p* < 0.05. As expected, combination of three US features outperformed those based on only one feature, and combination achieved the highest AUC of 0.782 and accuracy of 0.77 (Fig. 1). The maximum Youden index was obtained at a probability cut point of 0.468, yielding a sensitivity of 0.724 and a specificity of 0.744.

**Table 5:** Predictive value of ultrasonograhpic features.

Variables	Sensitivity	Specificity	PPV	NPV	Accuracy	AUC
Shape	0.303	0.851	0.26	0.876	0.426	0.577
Margin	0.652	0.246	0.25	0.831	0.439	0.449
Calcification	0.665	0.784	0.402	0.914	0.691	0.724
Shape + margin + calcification	0.724	0.744	0.485	0.853	0.770	0.782

**Figure 2:**
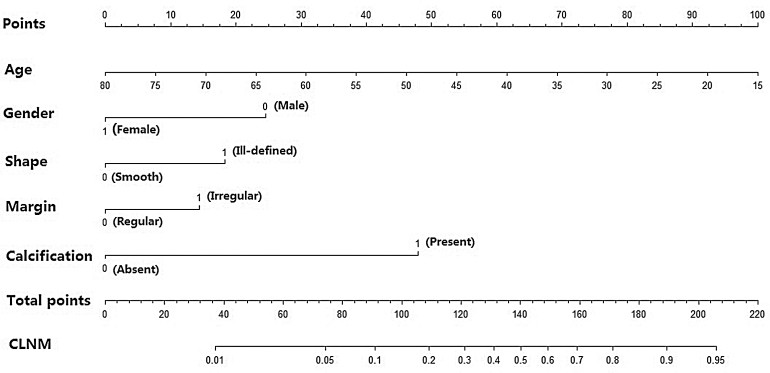
Nomogram for predicting CLNM in PTMC patients based on five risk factors.

**Figure 3:**
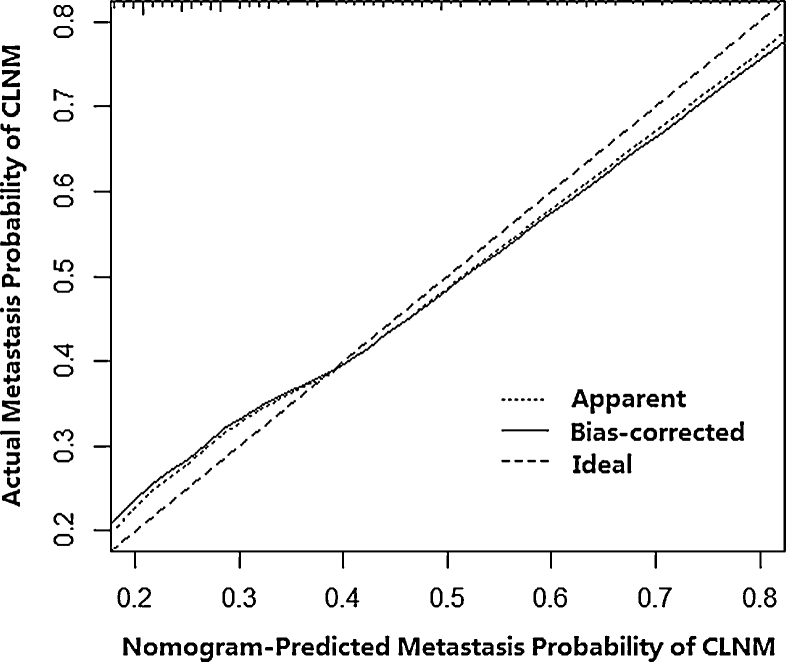
Calibration plots of nomogram for predicting CLNM (internal validation set).

Then, a predictive nomogram that incorporated the significant risk factors associated with CLNM was constructed based on the multivariate logistic regression model (Fig. 2). This nomogram integrated five risk factors (gender, age, shape, margin, and calcification) to assist in predicting CLNM in PTMC patients before the surgery. Each level within variables was assigned a score according to the point scale. By adding the total score and locating it on the total point scale, a corresponding probability of CLNM of each individual was determined. This nomogram had a good C-index of 0.835. The calibration plot also presented good agreement between nomogram-predicted metastasis probability and actual metastasis probability of central lymph node in Fig. 3 (mean absolute error = 0.019).

## Discussion

The reported incidence of PTC has increased steadily over time, predominantly because of increased detection of PTMC, and this will likely draw continued attention around the world. Despite its overall excellent prognosis, some PTMC may be aggressive and therefore also lethal because of distant metastases. Some studies reported that the rate of mortality in the first 10 years after surgery is <1%, and the locoregional disease recurrence in the same period is expected in 5% of patients.^19^, ^20^ Like PTC, PTMC has a relatively high frequency of CLNM, and the amount of CLNM might affect these patients’ prognosis. CLNM prevalence ranged between 13.0% and 64.1% in previous studies.^21^, ^22^, ^23^, ^24^, ^25^ We assumed the reason may be the heterogeneity in surgical technique, especially in the setting of CLND in which the extent of surgery may vary. In our study, the incidence of CLNM in PTMC was 22.4%, which was in accordance with previous reports. CLNM is an important risk factor of recurrence, and is not often detected clinically in patients. Thus, identification of predictive factors associated with CLNM may guide appropriate surgical strategies for patient.

We found PTMC had a higher rate in female patients (82.5%), and CLNM rate is higher in male patients (37.1%). The results demonstrated that maleness was an independently predictive factor of CLNM. We agreed with the explanation which might be associated with the fluctuation of sex hormone during a female's menstrual cycle and pregnancy.^26^ For the male patients, the normally higher basal metabolic rate might incite an overactive proliferation of tumor cell, and it lead to more metastasis. Previous reports have indicated multifocality as an independent risk factor for CLNM, and we tested it in this study. The incidence of CLNM in patients with multifocal PTMC was 29.3% as compared to 20.3% in patients with unifocal disease. We found multifocality was associated with a significantly increased risk of CLNM. It remains uncertain whether the de novo occurrence of distinct tumors or multiple foci in PTC origin from a single intrathyroidal tumor. Some evidence has suggested that noncontiguous tumor foci originated from independent precursors.^27^ Jovanovic's study found that 83% of multiple foci in PTMC had genetic alterations consistent with a monoclonal origin based on genome-wide allelotyping which indicated that monoclonally derived and multiple foci developed through the intrathyroidal metastasis from a primary tumor.^28^

Age is considered an important prognostic factor for survival in patients with PTC >1 cm; however, its prognostic value in PTMC is uncertain with inconsistent results.^29^, ^30^, ^31^ The AJCC/UICC TNM system uses the age of 45 years as a cutoff for upstaging patients.^32^ In this study, we found the rate of CLNM was higher in patients <45 years than that ≥45 years (31.0% vs. 14.6%), and age was an independent predictor of CLNM by univariate and multivariate analyses. We assumed that younger patients probably have more aggressive features than older patients. In clinical practice, more careful preoperative assessment of the lymph nodes status must be followed in young patients, and the treatment strategy for young patients might be different from that for older patients. For example, CLND may be better for younger patients, whereas observation may be best for old patients.

The risk of CLNM is known to increase with tumor size. Previous studies have suggested that tumor size is an independent predictor of CLNM,^5^, ^23^, ^30^, ^31^ but the cutoff size differed across various studies. Although the ideal cutoff value of size for the threshold of PTMC is still uncertain, most studies use 0.5 cm as the size threshold and have investigated the aggressiveness of PTMC. They proposed PTMC >0.5 cm was more significantly associated with CLNM than those <0.5 cm. The result in this study showed that more CLNMs could be found in PTMC >0.5 cm; however, it failed to reach statistical significance in the univariate analysis. The rate of extrathyroidal invasion for PTMC varies a lot from different studies (2%–21%).^24^, ^32^ The difference in the incidence might be accounted for by different diagnostic criteria. Some studies considered extrathyroidal invasion only when gross invasion into extrathyroidal structures occurs, while others included both microscopic and extensive extrathyroidal invasion. In our study, extrathyroidal invasion was demonstrated as an independent clinicopathologic factor for predicting CLNM. Although we cannot separately analyze the two subtypes of extrathyroidal invasion, some studies have shown that the different subtypes might represent a similar predictive value for CLNM in PTMC.^4^, ^7^

It is known that the occurrence of HT is higher in PTC patients.^1^ Some previous studies reported that coexisting HT in PTMC was correlated with LNM.^33^, ^34^ However, in a PTMC report, So et al. found 24.9% of PTMC patients had HT and there was no association between HT and LNM.^29^ Kim et al. also reported that HT did not affect the frequency of LNM in PTMC.^33^ In this study, the rate of HT was 19.9% in PTMC patients, and the rate of CLNM was 25.2% in the HT group, which was higher compared with non-HT group (21.7%). However, there was no association between HT and CLNM in this study. Currently, the effect of coexistent HT on prognostic outcomes in PTMC is still unclear.

At present, ultrasonography is widely used to evaluate CLNM in PTMC patients, both for the initial staging and during the subsequent surveillance following thyroidectomy. However, it has a poor diagnostic capacity for CLNM because it is affected by gas, bone and glands. Some studies have addressed the wide variability in sensitivity (10.9%–94%) and specificity (69%–90%) of US in detecting CLNM.^35^, ^36^, ^37^, ^38^ In our study, US had high specificity (83.2%) and low sensitivity (20.1%) in diagnosing CLNM, which was similar to those of previous studies. The high rate of CLNM and low sensitivity of US make it challenging to investigate which factors are associated with CLNM. Previous studies have identified some typical US features of LNM in PTC, including microcalcification, intranodal cystic component, round or taller-than-wide shape, and irregular border. Similar results were found in this study, and it revealed that shape, margin, echogenicity, calcification, and A/T were risk factors in univariate analysis. However, multivariate analysis demonstrated that only shape, margin, and calcification were independent factors.

The number of primary nodules is one of the factors used to assess the prognosis of PTMC. However, our result showed that multifocal nodules were not significantly related to CLNM. It may have been due to the high incidence and deceptive US images of benign thyroid nodules. Based on pathological results, we found that multifocality increased the risk of CLNM. Thus, if all the patients with multifocal tumors had preoperative US and US-guided FNAB, it might improve the judgment of the lesions, and this finding might has been different. Some studies had reported that the incidence of CLNM was related to the location of PTMC.^23^, ^39^ Our results showed that nodule located in the lower pole of the thyroid gland had higher rate of CLNM. We postulated that tumor cells from the mid-lower region of thyroid gland might be more likely to be transported to the tracheoesophageal groove in the central lymph nodes due to the nature of the lymphatic drainage system of the thyroid gland.

Calcifications in the metastatic lymph nodes are the focal accumulation of calcium salt, and part of the mechanism may be the rapid proliferation of tumor cells and hyperplasia of cancer tissues.^36^ In this study, the presence of calcification was found to be a typical sign that suggested cervical lymph node involvement. Rosario et al. proposed that calcification had a specificity and positive predictive value of 100%, because it was not observed in any normal or reactive lymph nodes.^40^ In the 2015 ATA, microcalcificaiton has been regarded as one of the highly specific signs and important US findings of suspicious malignant nodules. In addition, some authors reported that dense vascularity was associated with high blood flow velocity, and was related with rapid tumor growth nourished by rich blood from new vessels, leading to larger contact areas for cancer and lymphatic vessels, which might result in LNM.^16^ However, blood flow in our study was not a predictive factor for CLNM.

US evaluation is operator-dependent and no single feature is adequately sensitive for detection of CLNM in PTMC. Although many studies have described preoperative US features of PTMC and identified several predictive factors, few of them integrated all risk factors for prediction of CLNM. In this study, we found the combination of three US features (shape, margin, and calcification) showed superior performance in predicting CLNM compared with other single factor. The sensitivity, specificity and accuracy were increased respectively to 72.4%, 74.4% and 77.0%. Using ROC curve, we identified 0.468 as the cut-off value for predicting CLNM with PPV of 48.5% and NPV o 85.3%. In addition, we also developed a nomogram based on the identified risk factors because a nomogram could integrate all of these variables together and give an individual estimation of the metastatic risk for each patient. Nomogram has been reported to be helpful in dealing with dilemmas in breast cancer, but it has been rarely reported for predicting CLNM risk in PTMC patients.^41^ To use this nomogram, a PTMC patient's value was located on the corresponding variable axis, and a vertical line was drawn upwards to acquire the number of total points. All of the points were then added, and then the sum was located in the CLNM risk axis. Thus, we could obtain the estimated likelihood of CLNM in every PTMC patient. Our nomogram showed a good discriminative ability, with a C-index of 0.835. We consider that it is a reliable tool to help clinicians to decide whether to perform CLND in PTMC patients.

We acknowledge that there are several limitations in our study. First, it is a retrospective study that focused on data from a single center, which may had resulted in selection bias. Second, the sample size was not large enough, with only 599 patients being ultimately identified. Larger sample size studies are needed to further confirm our predictive model. Simultaneously, the mean follow-up duration of 44.28 months seems to be short considering the natural course of thyroid cancer, and it could increase the statistical uncertainty. Lastly, the nomogram needs further validation in a large prospective cohort of PTMC patients.

## Conclusion

In summary, clinicopathologic factors of male gender, younger age (<45 years), multifocality, extrathyroidal invasion, and LLNM are independent risk factors predicting CLNM. We conclude that CLNM may be closely associated with US characteristics including irregular shape, ill-defined margin and calcification, implying that PTMCs with these US appearances are likely to have a higher risk of CLNM. Clinicians should pay close attention to the patients who have these predictors. We generated a nomogram model for predicting CLNM risk in PTMC patients. It incorporated five independent variables and showed a good predictive accuracy. Preoperative evaluation and pCLND may be indicated when the patients have a high nomogram score.

## Funding

This work was supported by the Natural Science Foundation of Liaoning Province (No.201602450, No.20180530038).

## Conflicts of interest

The authors declare no conflicts of interest.
